# Impact of humanized APOE on brain volumetrics and white matter microstructure within a mouse model for Alzheimer’s Disease

**DOI:** 10.1002/alz.092751

**Published:** 2025-01-09

**Authors:** Avnish Bhattrai, Adam C. Raikes, John W. McLean, Jean‐Paul L. Wiegand, Roberta Diaz Brinton

**Affiliations:** ^1^ University of Arizona, Tucson, AZ USA

## Abstract

**Background:**

Frequently utilized Alzheimer’s disease (AD) preclinical models rely on risk factors expressed in familial AD, which accounts for <1% of the clinical AD population. Apolipoprotein (APOE) ε4 is the strongest genetic risk factor for the development of the more prevalent late‐onset Alzheimer’s disease (LOAD). MRI studies demonstrate a link between APOE‐ε4 and reduced gray matter volume as well as lower fractional anisotropy (FA) in AD patients. However, brain volumetric and white matter integrity (WMI) data in preclinical models is lacking. Herein, we report the effect of sex and genotype on local brain volume and WMI in an aged humanized APOE (hAPOE) LOAD mouse model.

**Method:**

Aged hAPOE ε3/3, ε3/4, and ε4/4 mice (N=43, mean age=24.1 months, Female=20) underwent high‐resolution ex‐vivo structural (T2‐weighted RARE; 75 microns isotropic) and diffusion‐weighted (b = 1000 mm/s2; 200 microns isotropic) MRI imaging. Deformation‐based morphometry was used to identify volumetric differences and WMI was quantified using FA. Mice were classified as ε4 carriers (at least one allele) or non‐carriers (ε3/ε3). Two‐way ANOVAs were used to identify the main effects of ε4 genotype and sex. Results are reported as FDR corrected. p≤0.05 was considered statistically significant.

**Result:**

hAPOE‐ε3/ε3 mice had larger olfactory bulbs and parieto‐temporal lobes compared to ε4 carriers and greater mean FA across major white matter tracts. Independent of genotype, females had larger cortical volumes of bilateral temporal and parietal lobes and thalami compared to males. Males had greater volume within the bilateral olfactory bulb, amygdala, hypothalamus, and hippocampi. No sex differences were observed in FA.

**Conclusion:**

At 24 months of age (approximately 70 human years), hAPOE ε4 mice exhibited decreased WMI compared to ε3s. Decreased WMI in ε4 carriers is consistent with recent work linking APOE with reduced myelin in the APOE ε4 human brain. Further, sex differences select brain region volumes indicate sex‐specific patterns of aging in this model. Collectively, outcomes of these analyses indicate both sex and APOE genotype differences in both gray matter volumes and in white matter integrity.

